# Homozygous DNAJC6 Mutated Juvenile Onset Dystonia‐Parkinsonism Is Responsive to Pallidal Deep Brain Stimulation

**DOI:** 10.1002/mdc3.14300

**Published:** 2024-12-10

**Authors:** James Manfield, Marko Bogdanovic, Nagaraja Sarangmat, Sangeeta Scotton, Alexander L. Green, James Fitzgerald

**Affiliations:** ^1^ Oxford Functional Neurosurgery John Radcliffe Hospital Oxford UK; ^2^ Department of Neurology Royal Wolverhampton Hospital Wolverhampton UK; ^3^ Nuffield Department of Surgical Sciences University of Oxford Oxford UK

**Keywords:** DNAJC6, dystonia‐parkinsonism, DBS

Dear Editor,

DNAJC6 gene mutation is a recently discovered^1^ autosomal‐recessive cause of Parkinson's disease. Several phenotypes are described including slowly progressive levodopa‐responsive parkinsonism with motor fluctuations and dyskinesia; rapidly progressive parkinsonism with intellectual disability, cognitive decline, upper motor neuron signs, dystonia and epilepsy; and dystonia‐parkinsonism.^2^ Most reported cases are of juvenile onset and despite multidisciplinary input satisfactory symptomatic management remains challenging.^2^, ^3^ We describe here a case of juvenile onset disease due to a newly described genetic mutation which demonstrated excellent response of dystonia to bilateral pallidal deep brain stimulation following exhaustion of pharmacotherapy.

A 19‐year‐old Caucasian female was referred for a surgical movement disorder multidisciplinary opinion. She was adopted aged 3 following neglect. She attended mainstream school with normal development, including motor function, speech, and cognition, until aged 13. Then she started tiptoe walking and unprovoked vomiting. This was followed by language regression, reduced food intake, weight loss and tremor. Initial diagnoses of anorexia nervosa, anxiety, pervasive arousal withdrawal syndrome and functional neurological disorder were considered until more classical parkinsonian signs emerged later in her teens. These progressed with marked difficulty in walking and turning, a generalized tremor of all limbs and drooling, necessitating 24‐hour care. Distressing dystonic features then predominated, comprising daily dystonic storms (retrocollis, throat constriction and oculogyric crises (OGCs)—see Videos 1 and 2). Communication also regressed requiring Makaton adoption and specialist schooling. A jejunostomy became necessary for progressive gastroparesis and pseudo‐obstruction.

**Video 1:** Shows sustained oculogyric crisis and dystonic posturing causing evident patient distress.

**Video 2:** Shows the dystonic retrocollis also seen prior to DBS surgery.

Comprehensive biochemical investigations were performed on serum, urine, and CSF, excluding a metabolic or neurotransmitter disorder, with MRI and spectroscopic imaging also being unremarkable. Whole genome sequencing revealed homozygosity for a DNAJC6 gene pathogenic variant confirming a genetic diagnosis of juvenile onset dystonia‐parkinsonism. This variant (see Table 1) introduces a premature stop codon triggering nonsense mediated decay in a gene where loss of function is a known mechanism of Parkinson's disease 19a.^3^ This constitutes very strong evidence (PVS1) of pathogenicity as per consensus criteria^4^, ^5^ and represents a novel variant not hitherto reported.

**TABLE 1:** Genetic variant classification (ACMG/ACGS guidelines)^4^, ^5^

Nomenclature	DNAJC6 (NM_001256864.1): c.1478C > A p.(Ser493Ter)
Classification	Pathogenic
Zygosity	Homozygous
Inheritance	Unknown

Treatment with levodopa and ropinirole was initiated with partial motor symptom response, but her oculogyric and dystonic crises became more frequent and severe until multiple were happening daily (See Videos 1 and 2). Limited benefit was obtained from trials of anticholinergic and benzodiazepine agents including escalating buccal midazolam dosing. With pharmacotherapy exhausted, and dystonic crises necessitating hospital admissions, palliative bilateral posteroventral GPi deep brain stimulation was offered. This was performed uneventfully with direct imaging‐based targeting under general anesthesia. Bilateral directional leads (Boston Scientific Cartesia) were inserted and connected to an infra‐clavicular implantable pulse generator. At 6‐months follow up, her OGC and stereotypies had virtually resolved with only two mild and self‐terminating episodes since surgery. There was also significant benefit in both tremor (now minimal—compare pre‐op Videos 3 and 4 with post op Videos 5 and 6) and vomiting, resulting in an overall dramatic improvement in quality of life. There was slight improvement in gait and no change in dysphagia. Overall UPDRS Part III scores improved from 87 pre‐op to 55 at 6 months reflecting improvements in both tremor and bradykinesia. BFMDRS movement scores improved slightly (57 pre‐op, 54 post‐op), but do not capture the main benefits of dystonic crises resolution. Her medication burden was gradually reduced with successful withdrawal of levodopa and benzodiazepines (clobazam and midazolam).

**Video 3:** Shows pre‐operative upper limb clinical examination with tremor and bradykinesia apparant.

**Video 4:** Pre‐operative lower and upper limb exam.

**Video 5:** Post‐operative upper limb clinical exam showing improvement in tremor and bradykinesia.

**Video 6:** Post‐operative lower and upper limb exam.

DNAJC6 encodes the brain‐specific isoform of Auxilin, which has an established role in Clathrin mediated endocytosis. This is an important mechanism for new vesicle formation at the presynaptic terminal and synaptic vesicle recycling, dysfunction of which is believed to underly the genetic alteration's pathological effects.^6^ Reported DNAJC6 mutated phenotypes broadly comprise either neurological regression with movement disorders, seizures, GI dysfunction etc., or early‐onset Parkinson's disease in third to fourth decades. DBS has been described in the latter group but not the former as reported here. A review of DNAJC6 mutations in early onset PD included two adult patients treated surgically, one with bilateral STN DBS and the other with pallidotomy. The results were apparently good, but the patients were phenotypically different than here, with both developing classical levodopa‐responsive PD symptoms in their 30s.^6^ Nevertheless, given the evidence of efficacy in some other monogenetic PD disorders,^7^ a reasonable prospect of benefit was hypothesized and surgery offered due to the profound distress caused by daily dystonic storms refractory to medical management. The GPi was selected as the target due to dystonic rather than parkinsonian features being most intrusive. At six‐month follow up, the greatest benefit was seen in the dystonic manifestations, with resolution of retrocollis and OGCs, but there was also worthwhile improvement in PD manifestations, particularly tremor and rigidity. It is noteworthy that this DBS benefit exceeded the degree of pre‐operative levodopa responsiveness. Indeed, the partial pre‐operative response to levodopa seen here is consistent with variability reported in other juvenile onset DNAJC6 PD patients. Adult‐onset cases, in contrast, are typically levodopa responsive with a more classical PD phenotype. Whilst the impact of DBS on the longer‐term clinical course remains unknown, based on this experience DBS can be effective in juvenile‐onset DNAJC6 dystonia‐parkinsonism and its use may be considered in medically refractory cases by managing multi‐disciplinary teams.

## Author Roles

Manuscript Preparation: A. Project conception B Writing of the first draft, C. Review and Critique.

J.M.: 1A, 1B.

M.B.: 1C.

N.S.: 1C.

S.S.: 1C.

A.G.: 1C.

J.F.: 1A, 1C.

## Disclosures

**Ethical Compliance Statement:** Formal ethical approval was not required for this retrospective case report. Written informed patient consent was obtained and documented on a standardized consent form. We confirm that we have read the Journal's position on issues involved in ethical publication and affirm that this work is consistent with those guidelines.

**Funding Sources and Conflicts of Interest:** No specific funding was received for this work. The authors declare that there are no conflicts of interest relevant to this work.

**Financial Disclosures for the Previous 12 Months:** ALG reports consulting fees from Abbott, InBrain and is a founding member of Amber Therapeutics. JF reports advisory board, consulting fees and educational honoraria from Abbott, consultancy for Eli Lilly and research grants from UCB and Merck. The remaining declare that there are no additional disclosures to report.

## Data Availability

Data sharing is not applicable to this article as no new data were created or analyzed in this study.
